# Key Considerations on CITE‐Seq for Single‐Cell Multiomics

**DOI:** 10.1002/pmic.202400011

**Published:** 2025-02-09

**Authors:** Hye‐Wong Song, Jody Martin, Xiaoshan Shi, Aaron J. Tyznik

**Affiliations:** ^1^ Single‐Cell Multiomics Team BD Biosciences San Diego California USA; ^2^ Applied Research & Technology, Medical Scientific Affairs BD Biosciences Milpitas California USA; ^3^ Applied Research & Technology, Medical Scientific Affairs BD Biosciences San Diego California USA

**Keywords:** antibody‐derived tags, cellular indexing of transcriptomes and epitopes by sequencing, library preparation, next‐generation sequencing, proteogenomics

## Abstract

CITE‐Seq (Cellular Indexing of Transcriptomes and Epitopes by Sequencing) is an advanced single‐cell sequencing method to profile both gene expression and protein abundance simultaneously in individual cells using single‐cell mRNA sequencing techniques alongside antibody‐derived tags (ADTs), for protein detection. The characterization of both the transcriptome and the proteome from the same cells provides a powerful and multiomic approach for understanding the mechanisms of complex biological processes. This review focuses on the workflow of CITE‐Seq using a microwell‐based single‐cell analysis system as an example and provides key considerations for staining cells with ADTs. By highlighting critical information for CITE‐Seq library preparation, sequencing, and data analysis, this review provides a practical guide with which to perform comprehensive CITE‐Seq workflow.

**Abbreviations**
ADT: antibody‐derived tags
CITE‐Seq: Cellular Indexing of Transcriptomes and Epitopes by Sequencing
NGS: next‐generation sequencing
scRNA‐seq: single‐cell RNA‐seq
UMI: unique molecular index

## 1 Introduction

The development of commercial solutions for Cellular Indexing of Transcriptomes and Epitopes by Sequencing (CITE‐Seq) technology has enabled the broad scientific community to better understand the rich diversity of cell types and to characterize the heterogeneity of different disease states [1, 2, 3]. CITE‐Seq allows simultaneous quantification of both protein and mRNA at the single‐cell level, providing greater potential to uncover cellular diversity compared to uncoupled, single‐modality approaches such as single‐cell RNA‐seq (scRNA‐seq) or single‐cell proteomics [4]. The ability to examine protein levels directly using CITE‐Seq is useful for cellular phenotyping in situations where mRNA is not well correlated with protein expression [5], post‐transcriptional changes are critical to capture or when mRNA transcript level is low. For example, CITE‐Seq utilizes high‐affinity antibodies to specific protein epitopes, which may detect isoforms such as CD45RA and CD45RO, offering a solution to overcome the inherent limitations of single‐cell transcriptomics [6].

**FIGURE 1:**
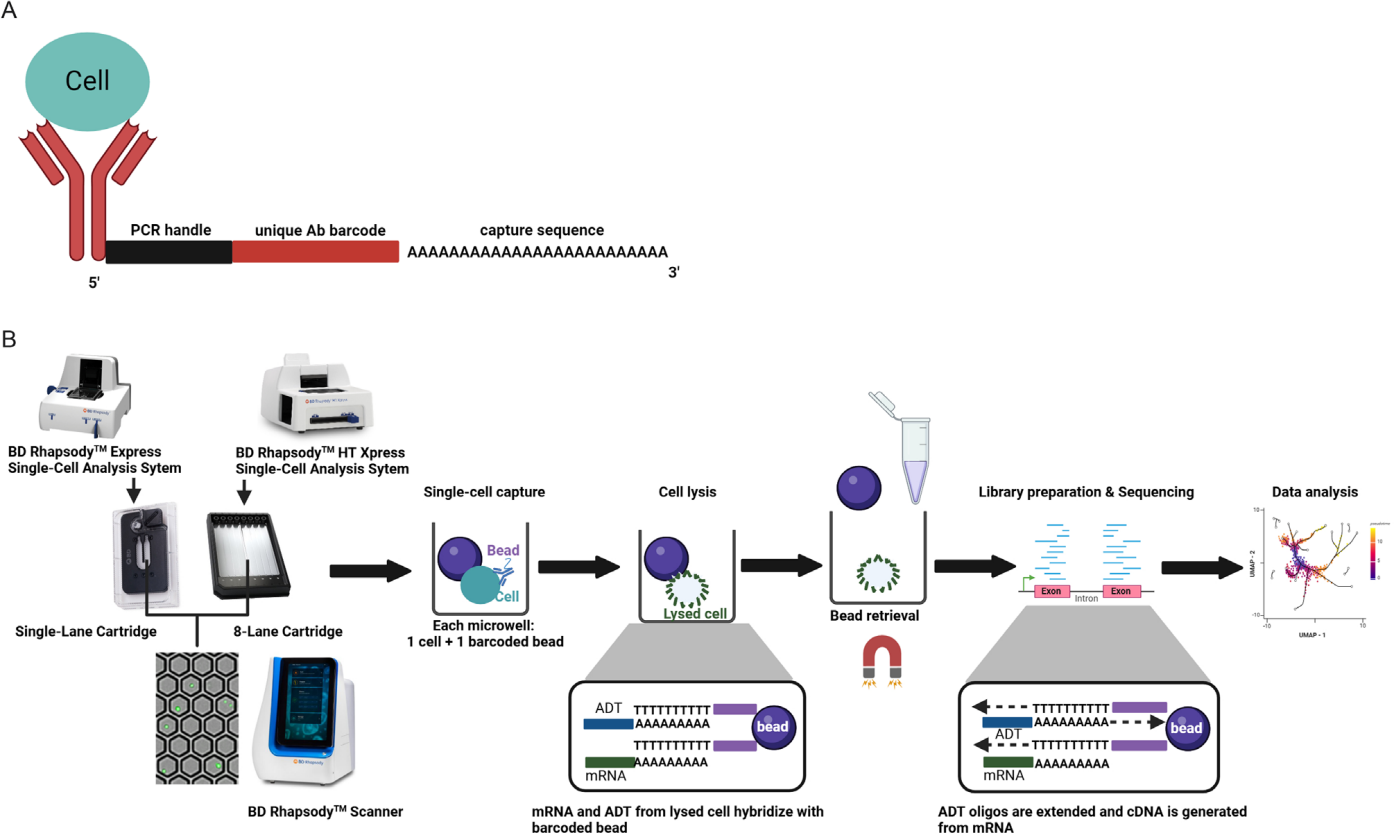
Workflow of CITE‐Seq using microwell‐based single‐cell analysis system. (A) Structure of antibody‐derived conjugate. ADT is an antibody that is conjugated with an oligonucleotide sequence. From the 5′ to 3′ end, the oligo includes a PCR handle that can be amplified during library preparation, a unique antibody barcode, and a capture sequence. For BD AbSeq Reagents, the capture sequence is a stretch of adenine nucleotides that can be captured using BD Rhapsody^TM^ Beads. (B) Single‐cell capture workflow using the BD Rhapsody^TM^ System. Single‐cell suspensions are first loaded into the BD Rhapsody^TM^ Cartridge containing microwells. Both single‐lane cartridges (used with the BD Rhapsody^TM^ Express Single‐Cell Analysis System) and 8‐lane cartridges (used with the BD Rhapsody^TM^ HT Xpress Single‐Cell Analysis System) are available to capture either one sample or up to eight samples simultaneously, respectively. Cells can also be stained with cell stain, Calcein AM, and dead cell marker, DRAQ7, prior to cell loading to visualize using the image‐based quality control instrument BD Rhapsody^TM^ Scanner. Scanner metrics help confirm the cell viability until cells are lysed and the success of each step of the single‐cell capture workflow. After cell loading, BD Rhapsody^TM^ Beads with unique cell labels are loaded into microwells so that each cell can be captured by a bead. After cell lysis, mRNA and ADT oligo are hybridized with the BD Rhapsody^TM^ Bead in each microwell. Beads are then retrieved using magnetic force and processed for downstream reverse transcription. During the reverse transcription, ADT oligos are extended in both directions and cDNA is generated from mRNA. Parallel mRNA and ADT libraries can then be generated using a BD Rhapsody^TM^ Assay Kit prior to sequencing.

The key reagent in the CITE‐Seq workflow is an antibody‐oligo conjugate [7]. The single‐stranded DNA‐oligo sequence contains a PCR handle, unique antibody barcode, and a generic sequence such as a polyA tail that can be captured during the single‐cell partitioning and lysis steps of a single‐cell multiomic workflow (Figure 1A). This review utilizes the BD Rhapsody^TM^ Single‐Cell Analysis System as an example to illustrate the CITE‐Seq workflow; however, other single‐cell capture systems such as the droplet‐based system are also adopted in the field. Experimental considerations discussed here are applicable to different single‐cell capture systems.

The BD Rhapsody^TM^ System leverages a microwell‐based partitioning system to enable measurement of both gene and protein expression with single‐cell resolution. The technology was initially developed in 2015 [8] and makes use of an array of microwells for single‐cell isolation and a diverse pool of barcoded beads to capture nucleic acids such as mRNA as well as ADTs. Briefly, the BD Rhapsody^TM^ System workflow starts by loading single‐cell suspensions into a cartridge with microwells (Figure 1B). Based on Poisson distribution, a majority of cells will occupy a unique microwell in the cartridge with high probability. If an increased number of cells is loaded, more multiplets will occur but can be partially removed during bioinformatic analysis. Following the addition of excess barcoded capture beads, most wells will contain one bead and, importantly, most cell‐containing wells are paired with a single bead. After cell lysis, mRNA and oligos from ADTs are released and hybridize to complementary oligos on the bead (Figure 1B). Beads can be pooled and retrieved using a magnet and then processed for cDNA synthesis. After cDNA synthesis, cDNA as well as the complementary sequence of the ADT oligos will be covalently bound to the beads which can be stored for as long as 1 year [9]. With downstream PCR, both ADT and mRNA libraries can be amplified and then separated for index PCR during library preparation and sequencing [10].

## 2 Cell Staining With ADTs

### 2.1 Cell Staining With Only ADT Reagents

General considerations for cell staining with antibodies apply to staining with ADTs as well. Cell viability is of great importance for ADT staining. Dead cells in the sample will lead to nonspecific binding of ADT reagents, reduced efficiency of cell partitioning, and compromised mRNA integrity for scRNA‐seq in CITE‐Seq workflow [11]. For cell samples containing myeloid and/or B cells, addition of an Fc‐block reagent before ADT staining is recommended to possibly reduce Fc receptor‐mediated nonspecific binding [12]. Based on the cell type, users may choose to stain cells at either room temperature or at 4°C to help maintain a viable state [13]. In addition, evidence from the field of flow cytometry suggests that some antibody targets may benefit from a pre‐incubation at 37°C; an example is chemokine receptors [14]. After staining, sufficient cell washing is needed to reduce noise associated with residual and unbound ADTs that may be captured in droplets or microwells. However, an increased number of cell washes can lead to cell loss and different levels of cell loss may occur to different cell types in a heterogeneous biological sample [15].

**FIGURE 2:**
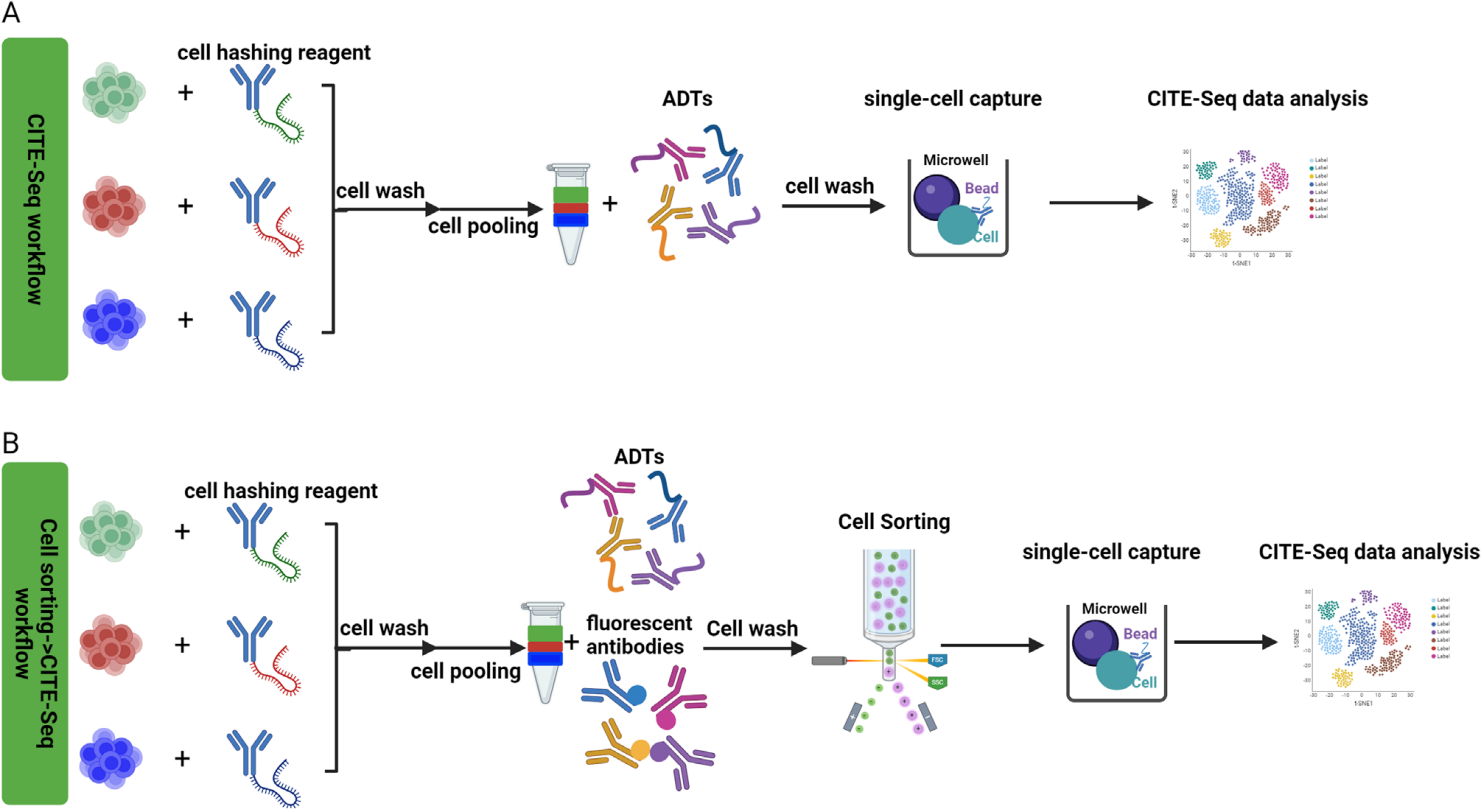
Workflow of cell staining prior to CITE‐Seq. (A) An example of cell staining workflow for CITE‐Seq. Users can stain different samples with cell hashing reagents and then pool them together for ADT panel staining prior to single‐cell capture. (B) An example of cell staining workflow followed by cell sorting for CITE‐Seq. If cell sorting is needed to enrich the cell population of interest, users can co‐stain cells with both a fluorescent antibody panel and ADT panel and then perform cell sorting before single‐cell capture.

Alternative ADT reagents, such as cell‐hashing antibody‐oligo conjugates, can be utilized in the same staining protocol as CITE‐Seq ADTs for sample labelling (Figure 2A) [16]. Cell hashing reagents usually bind with cells in each sample, allowing for multiple samples to run in one lane and to deconvolute each sample from the pooled sample bioinformatically, reducing batch effects between runs and leading to better identification of multiplets [17]. Different types of cell hashing reagents are available to accommodate different CITE‐Seq experimental designs: they can target a universal cell surface marker [18] or nuclear membrane proteins for labelling isolated nuclei [19] or integrate with the plasma membrane of cells or nuclei [20]. It is worth noting that different cell hashing reagents may have different binding efficiencies in labelling target cells. Users need to select the best option for their experimental design [20].

### 2.2 Co‐Staining With Both ADT Reagents and Fluorescent Antibodies

A major advantage of CITE‐Seq is its robust ability to broadly profile an array of cell populations at high resolution. However, this deep profiling on a large scale can increase experiment costs. To interrogate cell populations of interest and avoid sequencing unwanted cells, scientists often choose to isolate cell subsets of interest using a cell sorter and then process the sorted cells with downstream CITE‐Seq [21]. The sorting process also increases the relative cell number for rare subsets in a heterogeneous sample, allowing deeper interrogation of the rare cell populations [22].

To perform CITE‐Seq on sorted cells, the most efficient way is to stain cells with both fluorescent antibodies and ADTs simultaneously by co‐staining (Figure 2B) [23]. It reduces antibody incubation time and the number of cell washes compared to sequential staining, allowing improved cell integrity as well as minimizing cell loss through additional washes.

## 3 CITE‐Seq Experimental Design

### 3.1 ADT Titration

It is generally recognized that most assays using antibodies need reagent‐specific titration to achieve optimal results. Titration to identify the optimal concentration of ADTs for CITE‐Seq is especially important since high concentrations of ADTs can generate high background (mediated by non‐specific antibody binding) as well as sequester unwanted sequencing reads, which negatively impacts overall resolution. Several studies highlight the importance of ADT titration [24, 25, 26, 27, 28] even though variations in experimental design and sample type may warrant slight modifications to ADT optimal concentration.

Correctly titrated ADTs can still result in non‐specific background. This is because free‐floating ADT oligos in droplets or microwells can be captured and sequenced. The level of background noise can be different between different scRNA‐seq platforms [29] and those should be removed by bioinformatics tools to obtain meaningful CITE‐Seq data [30, 31].

### 3.2 Panel Design for CITE‐Seq Experiment

Because the ADT reagents are not fluorescently labelled, it is easier to increase the number of ADTs used in each experiment. This is an advantage over flow cytometry because sometimes inability to resolve spectrally overlapped fluorescent labels can limit the plex of the experiment [32]. Despite these benefits, successful ADT panel design requires consideration of several factors as below.

**FIGURE 3:**
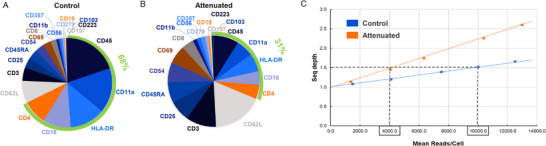
Better resolution of low expressors with signal attenuation. The impact of signal attenuation on the resolution of low‐expressed markers can be tested using a resting and activated PBMC model, comparing the attenuated data to non‐attenuated controls. PBMCs are stimulated for 24 h with CD3/CD28 Dynabeads^TM^ before staining with the 20 plex BD AbSeq antibody panel and performing the BD Rhapsody^TM^ System workflow. Reads distributions to each BD AbSeq ADTs of the 20 plex ADT panel from the sequencing result are displayed. (A) 68% of reads were devoted to the five highly expressed antibodies as shown in green bar around the pie charts. (B) The reads devoted to the five highly expressed antibodies are down to 31% after the five ADTs were 1:2 attenuated. (C) Graph showing the BD AbSeq ADT sequencing depth obtained from number of reads/cell. Data demonstrated that the same sequencing depth can be obtained from only 40% of reads when the high expressors are attenuated. Read redistribution triggered by signal attenuation led to a higher average sequencing depth (dotted line).

1. Antibody clone compatibility—It is generally understood in flow cytometry panel design that clonal compatibility should be considered. If the epitope targets of two antibodies are in proximity, one antibody might hinder the binding of another one when the two reagents are used conjointly [33]. For example, the majority of anti‐γδ TCR fluorescent antibodies do not detect the target cell populations when used in combination with other T cell markers, likely due to the incompatibility of clones [34]. Users need to avoid incompatible clones in the same ADT panel.
2. Sequencing cost—Since ADTs are detected by sequencing reads, it is important to allocate enough NGS (next‐generation sequencing) reads toward ADT libraries to gain meaningful data. The required reads for each experiment typically increase with the number of ADTs in the panel. Of course, the number of ADT oligos associated with any given cell depends on the abundance of the proteins targeted. Panels including several ubiquitously or highly expressed protein targets will require more reads to maintain resolution across all targets [24]. Because sequencing reads are limited and all ADTs share the allocated reads to the same library, reads associated with the background are essentially wasted, leaving less reads for real signal and reducing the assay sensitivity. Therefore, using high‐plex panels (greater than 100 ADTs) for proteogenomic screening has limitations as many targets are unlikely expressed and non‐specific ADT background is read‐additive [24].
  In most cases, targeted panels of 30–40 markers are more meaningful for any given sample type and minimize sequencing costs [24]. Panels thoughtfully designed to include sample‐specific phenotyping markers and targeted cell functional markers can provide deep insights for a particular biological system [35]. The difficulty in pre‐designing and cocktailing panels is to optimize each ADT marker for different sample types. It is preferred to optimize backbone panels for specific areas of biology and then to enable ADT drop‐ins for specific studies [36].

3. Signal attenuation—Because all ADT markers share sequencing reads in each CITE‐Seq assay, and the fact that protein expression can range dramatically, proteins with high antigen density will consume a disproportionate share of reads. This leaves less reads for low‐expression proteins (Figure 3A), resulting in both exorbitant sequencing costs and poor resolution of low expressors. Simply titrating down antibodies of high expression targets is not ideal since antibody labelling is most consistent and accurate when using saturated concentrations [37].

To better resolve low‐expressed proteins while controlling sequencing costs, we developed the signal attenuation assay. To reduce the effective oligo‐labels per antibody signal for high expressors, we mixed unlabeled antibody lacking an oligonucleotide with ADT reagents. By attenuating five antibodies against high expressors with 1:2 ratio (ADT: unlabeled Ab), the resolution of low expression targets was increased two‐fold (Figure 3A,B). Thus, the same sequencing depth can be obtained from only 40% of reads when the high expressors are attenuated (Figure 3C). Although it can be laborious to identify the ratio and antibody mixture, the benefit from the signal attenuation assay may justify the effort. If pre‐mixed antibodies for high antigen density targets are commercially available, the assay can be easily adapted.

### 3.3 Intracellular CITE‐Seq

Although most published CITE‐Seq studies demonstrate analysis of cell surface proteins, efforts to expand the assay to enable analysis of intracellular proteins, intracellular CITE‐Seq, are ongoing [38]. Poor correlation between mRNA and protein expression is often observed [39, 40], highlighting the complexity of translational regulation. Additionally, protein functionality is further controlled by intricate post‐translational modifications [41]. Intracellular CITE‐Seq workflows provide new and important information when used with surface protein and mRNA profiling.

1. Transcription factor detection—Expression of transcription factors is often tightly controlled since these powerful proteins regulate transcription of functionally connected proteins that determine specific cell lineages [42]. Although mRNA detection of transcription factors can serve as potential biomarkers, the turnover rate for such mRNA tends to be fast [43, 44], making it hard to detect them using scRNA‐seq alone [45]. Therefore, having transcription factor protein detection by intracellular CITE‐Seq can provide confidence and additional insights on top of surface CITE‐Seq.
2. Phosphorylated protein detection—Cell signaling pathways often utilize protein kinase/phosphatase reactions to cascade signals, and assays that measure phosphorylated proteins can elucidate a cell's functional state [46]. Although mRNA alone or surface CITE‐Seq cannot provide such information, intracellular CITE‐Seq brings this capability to single‐cell multiomic studies. Detecting proteins undergoing such post‐translational modifications along with deep phenotype analysis using surface CITE‐Seq can provide more comprehensive biological information on mechanisms of cell regulation [47].
3. Secreted protein detection—Cells communicate not only intracellularly but also intercellularly through secreted proteins [48]. Therefore, secreted protein profiling provides further insight to better understand complex biological processes at the individual cell level and informs organ‐level function [49]. Since the secreted proteins are released from the cells, it is difficult to identify single‐cell protein secretion dynamics. One common method to detect secreted proteins is to utilize a cell transport inhibitor to artificially keep proteins inside cells followed by intracellular antibody staining [50]. Another method is to directly capture secreted proteins at the cell surface using surface‐anchored capture antibodies and then to detect them using an antibody sandwich like other immunoassays [51]. This can be done, after optimized sample preparation using standard surface CITE‐Seq, although it requires specific antibody pairs in addition to ADTs [52, 53].

Although intracellular CITE‐Seq can provide valuable insights, it is a difficult assay since intracellular antibody staining requires cell fixation and permeabilization which negatively impacts RNA assay in general [54]. To overcome this, a low concentration of formaldehyde fixative and detergent was used to permeabilize nuclei in inCITE‐Seq [55] and NEAT‐seq workflows [56]. A reversible cross‐linking reagent, DSP/SPDP, can also be used for fixation in the workflow [38]. Another caveat of intracellular CITE‐Seq is the oligo‐mediated non‐specific binding, which took place after ADT oligo enters into intracellular space filled with the large amounts of nucleotides. To prevent oligo‐mediated non‐specific binding, dextran sulfate [55], or a high concentration of tRNA solution [38] was added into the stain buffer. The other study utilized a blocking buffer containing single‐stranded DNA‐binding protein and random oligos [56].

### 3.4 Library Preparation and Sequencing

Once ADTs are bound with cells, ADT signals can be captured using different single‐cell platforms. When using the BD Rhapsody^TM^ microwell‐based system, ADTs with polyA capture sequences bind to the poly dTs on the beads and gain the cell barcode by reverse transcription during the cDNA synthesis step. ADT DNA‐oligos also extend toward the bead, which generates continuous double‐stranded DNA for the full‐length of the bead oligo and ADT oligo (Figure 1B). Depending on the downstream assay combination, ADT library is made utilizing either or both strands of the double‐stranded DNA.

After the library is generated, there are several factors to consider in terms of proteogenomic sequencing strategies. Since the size of the ADT DNA library is generally shorter than the mRNA library, smaller fragments are preferentially clustered during the sequencing run in certain sequencing platforms [57]. This should be considered when pooling libraries together with mRNA library for a single run. An additional factor to consider is that if the sequencing cycle is long, the ADT library may be called as having overlapping read 1 and read 2. Furthermore, read 2 often extends into the polyA sequence on the opposite end of the read, which can impact the overall Q30 score. This outcome of a lower Q30 score is expected for platforms that require diverse nucleotide composition, but importantly, it does not reflect the quality of the ADT barcode and unique molecular index (UMI) sequencing that is needed for successful data analysis. In cases of high Single Nucleotide Frequency (SNF) reads due to the presence of polyA sequence, trimming may help to restore some reads [58].

As mentioned above, the total read amount is set and each ADT will share from the ADT read allocation. Therefore, the number of dedicated reads toward the ADT library should be calculated based on the panel size. Additionally, when balancing the proportion of reads to mRNA and ADT libraries, it is important to consider the dynamic range difference between protein and mRNA molecules when planning sequencing reads allocation and evaluating sequencing depth for each library [59, 60].

### 3.5 Data Analysis

When sequencing is complete, the output data are processed through a specific bioinformatics pipeline for each single‐cell platform. The pipeline run includes reference mapping to identify gene/antibody barcodes, cell calling based on the number of mRNA reads associated with each cell barcode, and molecular counting based on UMIs [61, 62]. Mapping antibody barcodes requires a reference file that contains antibody barcode sequences for all ADTs used in the panel [63]. If the reference file misses any ADT barcodes used, the pipeline cannot map the reads and the protein expression will be missing in the final output data. Additionally, the quality of reagents such as oligo purity matters [64]. For example, if antibody A has 95% of its own barcode and 5% contamination of antibody B oligo, with a vast dynamic range of protein expressions in any given cell, the contaminating oligo signal can be quite high, leading to data artifacts. NGS can be used to ensure the high purity of oligo sequences prior to building the ADT reagent.

During single‐cell analysis pipelines, putative cells are identified by the number of mRNA reads per cell‐capture bead, likely since multiomic pipelines evolved from scRNA‐seq assays [65]. However, with the continued expansion of multiomic approaches, it may be beneficial to call cells by protein expression as well, because RNA‐derived cell calling may miss important protein‐containing cells that have low mRNA content [66].

There are several secondary analysis tools that have been developed to delve deeper into CITE‐Seq data for various biological extrapolations. Common mRNA analysis tools such as Seurat pipelines are valuable CITE‐Seq tools [67]. However, one consideration with these approaches is that molecular counts of protein and mRNA have different dynamic ranges [59, 60] and normalization may be needed before integrative analyses [68]. Effective solutions tailored to CITE‐Seq data analysis are also available such as total VI [69], CiteFuse [70], and LinQ‐View [71]. These tools often provide streamlined frameworks for comprehensive CITE‐Seq data analysis including dimensionality reduction, cell clustering, cell type identification, and differential expression of RNA/ADT analyses. Through a single pipeline that jointly analyzes mRNA and protein abundances, these tools simplify CITE‐Seq data analysis that would otherwise be conducted in separate pipelines and require manual alignment. It is worth mentioning that users can analyze CITE‐Seq data using SeqGeq [72] or FlowJo software. Scripts are available to convert CITE‐Seq data into the format that is readable by the FlowJo software [73]. An additional tool is also available to plot CITE‐Seq dataset into a flow cytometry‐like visualization [74].

Depending on sample quality, staining procedure, and single‐cell platforms, CITE‐Seq datasets can have high background issues that need filtering before secondary analysis [27]. To avoid batch effects or inaccurate differential marker expression, methods for removing background noise should be considered [30]. Advanced computational tools are emerging that enable the correlation of multiple CITE‐Seq datasets [75], or correlation between parallel experimental techniques such as CITE‐Seq, flow cytometry, and mass cytometry [76], allowing scalable and robust data integration.

## 4 Future Perspectives

Full‐scale proteomics at a single‐cell resolution has been a dream of researchers utilizing flow cytometry, mass spectrometry, and other proteomic techniques. However, they have met challenges including throughput, resolution, and scaling the number of simultaneous markers analyzed. The CITE‐Seq workflow was first published in 2017 and there have been nearly 3000 publications citing the work as well as several expanding on the technology's capability. As single‐cell proteogenomic workflows move beyond surface proteins to include intracellular and even secreted proteins, fully integrated proteomics with transcriptomics is becoming more of a reality. Like flow cytometry, CITE‐Seq is limited only by the available antibodies qualified in the workflow. Although CITE‐Seq's inclusion of whole transcriptomics can support gene and protein expression discovery, there is a need for expanded antibody‐oligo content for CITE‐Seq to realize true single‐cell proteogenomics.

Certainly, the need for multiomic assays at single‐cell or even sub‐cellular resolution spans all areas of biology and disease. Most biological tissues are solid, and spatial architecture is often critical. Emerging spatial technologies are following single‐cell transcriptomics growth trajectory into multiomic capability and ubiquitous utility [77]. Moreover, other techniques and platforms look to augment CITE‐Seq workflows with the inclusion of genomic DNA insights [56, 78, 79].

With these multiomic workflows becoming commoditized, there will continue to be a reduction in cost and complexity. As affordability and ease improve, multiomics becomes broadly accessible and we will see a convergence of technologies for many biological studies. Routine multiomic assays offer a unique opportunity to explore cellular heterogeneity and allow an in‐depth understanding of cellular states, which is helpful for the identification of novel biomarkers and understanding the mechanism of disease.

## Conflicts of Interest

All authors are full‐time employees of BD Biosciences.

## Data Availability

The authors have nothing to report.
